# To Develop Health Education Tools for Nasogastric Tube Home Caring Through Participatory Action Research

**DOI:** 10.3390/healthcare8030261

**Published:** 2020-08-10

**Authors:** Fang-Suey Lin, Hong-Chun Shi

**Affiliations:** Graduate School of Design, National Yunlin University of Science and Technology, Yunlin 64002, Taiwan; linfs@yuntech.edu.tw

**Keywords:** participatory action research, nasogastric tube, home care, medical graphics, PDSA, health education tools development

## Abstract

Medical institutions provide guidance on caring skills for home caregivers. Oral teaching is combined with graphical tools in a method that has been proved to be an effective way of quickly mastering home caring skills and promotes effective learning for home caregivers. The graphic design and operation contents of this method are constantly revised through interviews and observations, and by carrying out home care application graphics it forms a spiral structure of Plan–Do–Study–Act (PDSA) participatory action research (PAR). In the three cycles of the operation of PDSA PAR, the designers accurately create graphics of the caring details based on the nurses’ demonstrations and develop health education tools that are suitable to provide continuous assistance and services in real-life situations. PAR combined with PDSA, in each of the three cycles of the operation—design personnel, medical personnel and home caregiver personnel, respectively—as the lead roles, guide the planning decisions for PAR. This study is a reference for the improvement and development of medical graphics for health education tools to improve accuracy.

## 1. Introduction

Providing quality improvement training in nursing skills to home caregivers is essential for caring for patients at home, especially for those patients who require nasogastric intubation and catheterisation. Elderly or chronically ill patients are usually cared for by non-nursing professionals at home [1]. Most of the home caregivers in Taiwan are from Indonesia, Vietnam and The Philippines; how to effectively teach foreign home caregivers to take care of patients has become very important. Home caregivers have specific needs and challenges in the nursing environment, and their roles need to be recognised by the medical care system [2]. Home caregivers need to understand the symptoms and conditions of patients, soothe the patient’s emotions, be able to promptly and accurately report to the nurse in charge, have basic nursing knowledge and skills, and be able to communicate effectively [3,4,5]. In our research study, the nurses went to the patient’s home on average once a month; they replaced the patient’s nasogastric tube and guided the home caregivers in operating the tube in the home environment. A nasogastric tube is a type of medical instrument that intrudes into the body of the patient [6]. It is necessary to be able to carry out standard operation procedures of feeding, routine intubation cleaning, and position inspection as part of daily care.

Long-term nasogastric tube implantation easily causes infection. In addition to the nurses’ monthly professional nursing services, it can strengthen the caring procedures of home caregivers to receive relevant instruction in infection protection control, to avoid the infection of patients who are in the process of care [7]. Home caregivers should have relevant nursing knowledge and common sense when it comes to care.

The procedures of caring for a patient with a nasogastric tube are relatively complicated; nurses train home caregivers with the necessary caring skills mainly by demonstration, together with oral instruction. Due to the language barriers, the novice and foreign home caregivers are unable to communicate effectively and lack the relevant experience; these factors are likely to lead to problems occurring in the nursing process. Home caregivers who have direct contact with patients are the main providers of the patients’ needs. They have a responsibility to develop a shared experience with the nurses so that they can to better perform patient care services [8]. Home caregivers with nursing knowledge can provide better care for patients. Nurses need to take into account their level of understanding when communicating with home caregivers and exchange information with home caregivers effectively [9]. It is simple and inexpensive to use an image-based training tool to assist the users’ learning, which can improve their understanding and realise good learning results. Foreign home caregivers use graphical health education tools in the learning process, which is more convenient for the nurses’ teaching and the learners’ understanding. According to the research on the process of medical graphic intervention, the graphical operation steps can significantly improve memory and comprehension, help to correct faulty operation methods, and also avoid the boredom of traditional teaching methods [10]. The current clinical development process in graphics mostly considers the digital form but in the actual application, the final results of its use can be damaged by the lack of a corresponding evaluation process in the development process [11].

The current situation in home caregivers’ quality improvement training should be regarded as a concern of public health projects, which need to attach importance to nursing knowledge education and formulate relevant policies on the operational standards of caring work at home [12]. There needs to be development of the graphic design of the nasogastric tube home care process, with the participation of the designers, nurses, and home caregivers. Based on this joint cooperation and learning, the operation and instruction of nurses played a very important role in this research process. They assisted the designers in completing the development of designing the graphics and evaluated the home caregivers’ operation. The aims of this research are as follows:

- Using the participatory action research (PAR) method as the research tool, to design the graphics of procedures and considerations of nasogastric tube caring at home, explore a reasonable method of cooperation between design researchers and nurses, and develop a health education tool.
- The PAR in three cycles, in combination with the PDSA (Plan, Do, Study, Act) quality management steps and methods, continue to confirm the research objectives, repeatedly confirming and providing feedback on the contents of the graphics and texts, inspecting the quality of cooperation, and ensuring the accuracy and effectiveness of the process graphics drawing in the research process.
- The degree of participation, leadership, and knowledge exchange in each cycle by each of the participants are in different proportions; each participants’ position is revisited in the different cycles by the intervention of the researchers and the research tools. The relationships between nurses and home caregivers are transformed to improve the health education tools in the spirit of cooperation.

## 2. Literature Review

### 2.1. The Present Situation of Nasogastric Tube Home Caring

Although using a nasogastric tube may produce adverse effects, most families still choose to use one because it is convenient in clinical operation, can provide patients with nutrition, and patients have a chance of returning to normal eating. For patients with nutritional supplement, nurses prefer a nasogastric tube compared with other feeding methods; however, the nasogastric tube is not easy to operate [13]. According to the nursing profession, home caring should be divided into professional nursing operations and non-professional nursing operations. The use of home care is increasing because this method of care can save public resources [14]. The placement of a nasogastric tube requires specific operation by a professional nurse [15]. The lower the caregiver’s level of knowledge of the nasogastric tube care process, the higher the learning demand, and the corresponding evaluation should be included in the nasogastric tube operation process [16]. Nurses should assist home caregivers during home visits and, in general, provide intubation care manuals to improve the quality of care [17]. In addition to the guidance in intubation caring skills, nursing guidance is also required in good communication skills with which to communicate with the patients and to provide good care in terms of emotional comfort [18].

### 2.2. Participatory Action Research

Action research can transform knowledge into practice and then promote the formation of knowledge through practice, which is a process of continuous verification. Action research proposes changes based on the traditional collaborative approach and reflects on the whole research process, and importance is attached to the emotional feelings of all parties in the research process [19]. Action research is a process that combines theory and action to form a cycle of participation in practice. It is a process of co-creation, usually solving existing problems and then solving new ones. Different patterns and methods are adopted according to different professional studies [20]. Some action research does not presuppose the behaviour of the research object based on the theoretical framework but analyses and understands the research object by observation. Nursing is a patient-centred operation. In the context of nursing, action research can promote the coordination of the relationship between different participants [21]. Practical action research has a research cycle. To prove the validity of the theory, the practice process needs to have complete strategies and steps [22]. PAR is widely used in the field of sociological research, emphasising processes that are co-created by professional researchers and participants, including research agendas, methods, processes, and outcomes [23]. In the field of medical care, there are studies that intervene in nursing operations through PAR. Before that, nurses usually used personal experience to judge and were thought to have had communication with patients about medical behaviour. However, action studies in Intensive Care Units (ICU) have shown that encouraging nurses to be patient-centred from the perspective of patients’ problems—paying close attention to patients’ willingness to communicate—interferes with the communication behaviour of nurses, encouraging them to re-examine their relationship with the patient [24]. PAR often uses ‘plan–action–reflection’ in the process of nursing education; the adaptability and positive thinking ability of nursing students in clinical practice are thus improved [25]. The development of cross-disciplinary cooperation in the medical profession can develop common situational cognition. The action research method is the basis for this transformation, which integrates and transforms the knowledge of different specialties, changing the final practice through cooperation, communication, responsibility sharing, process improvement, and other methods [26]. PAR can promote the coordination of the interests of all the participants in the action research, give voice to patients, and take into account the equality of the interests of all parties in the research process [27].

This methodology has been applied to the knowledge to adapt the use of the PAR method to multidisciplinary cooperation, to enhance the understanding of the interdisciplinary knowledge domain, to create a knowledge spiral, and to put forward a proposal arising from the practice and put it into action such that the experts can arrive at a diagnosis [28]. However, this kind of knowledge spiral lacks the reflection process and may encourage participants to absorb knowledge in a passive state. PAR has many advantages but it can be problematic. The degree of participation of the participants varies, and the representativeness of the participants may not be widely extended to other group behaviours. This research method is considered to be unable to improve the power relationships among participants, and it is difficult to evaluate the effect of PAR on participation. Research claims are usually bold and important but avoid the negative effects and influences of excessive participation [29]. To generalise the results of the study to the benefit of other groups, communication and interaction with participants needs to be enhanced. To avoid the problem of excessive participation in PAR, selected participants have clearly defined roles to conform to the research aim and form a group of different participants, fully giving play to the role of individuals and group participants. The participants can comment at the same time, participate in the group of other individuals, give responses and supplements, and enhance participants’ confidence and motivation [30].

### 2.3. PDSA

PDSA’s approach is relatively easy to understand and its effects may be underestimated. PDSA operations with action cycles can give teams involved in an action a clearer direction [31]. PDSA can be used in a small range of operations to achieve good operational results. Service project improvement needs to be completed in different cycles [32]. PDSA should improve the problem step-by-step according to the cycles and be supported by sufficient data. Its own structure can promote the confidence of the team to deal with the problem, and the team will formulate a corresponding theory and obtain the result through appropriate observation and action [33]. Whether in the field of healthcare or other fields, the management framework using PDSA is to make planned changes and not deviate from the target in the improvement [34]. In the field of health profession education, it is believed that the application of the PDSA cycle can also promote quality learning, continuously construct the learners’ knowledge systems, and promote the generation of new knowledge [35]. The PDSA method is also used in the field of healthcare, and the method can be adopted for structured problem solving, promoting quality improvement [36]. According to the research-driven results, the research should make presuppositions or assumptions; and in the process of action research introspection, the train of thought should be continuously adjusted, improving study behaviour and ultimately affecting the results of the study; these results include cost, quality of nursing, and satisfaction, and there is continuous improvement in the quality of the research [37]. Moen & Norman (2009) introduced the development course of PDCA and PDSA during the development of PDSA, and various modified versions have been produced. The Shewhart cycle, considered to be the original version in 1939, established the three steps of specification, production and inspection. In 1951 Deming proposed an improvement cycle of four parts, namely design, product, sales, and redesign, after the Japanese Ishikawa PDCA cycle of problem solving corresponding to the four parts as follows: PLAN, corresponding to design: DO, corresponding to produce; CHECK, corresponding to sales; and ACTION, corresponding to redesign. This method emphasises that standards should be established and constantly revised before PDCA is implemented, and it is also commonly used in education and training. Deming believed that CHECK means preventing doing things and should emphasise the process of introspection and correction and generate the PDSA structure in later developments [38].

Although PDSA is considered to promote standardised operations and progressive learning in the field of nursing, PDSA does not solve all the complex problems. The application of PDSA in the nursing field is considered to be simple in operation and to lack scientific rigor. The application overemphasises individual ability while ignoring systematic problems. In practice, there may be problems such as adverse execution, so a clear and reasonable process is needed to implement PDSA [39]. To avoid invalid results in the PDSA operation process, researchers need to confirm the operability of the research problem. Full exchange of views and confirmation should be conducted among the participants to ensure the accuracy of the practical results. If the practice results are not effective, the introspection process needs to be carried out until the results are recognised by the professionals.

### 2.4. Application of Health Education Tool Graphic in the Caring Process

The improvement of medical and disease communication through nonverbal communication in the field of medical care has gained increasing amounts of attention. Graphical tools are mostly used in areas with different ages, low levels of health literacy, or insufficient cognitive ability so as to reduce the risks caused by cognitive or educational gaps. The graphics are applied to health communication. Spoken words or graphics can enhance the accuracy of the text, and the corresponding teaching methods can result in people focusing on the health materials. Graphics are especially useful for people with a low level of literacy. When graphics are applied in the field of health care, there should be corresponding evaluation [40]. Good health education and guidance can help to improve the problems in healthcare. Oral instruction can help to connect the graphics and words and can improve the care skills and confidence of caregivers, improve patients psychologically, and improve the communication between doctors and patients [41].

During the home care use of nasogastric tubes for the elderly, the main problems are that nurses and caregivers rarely use appropriate health education tools; there is no standardised process for the design of health education tools; health education leaflets lack design and publicity, and health education tools lack evaluation. This study aims to solve the problem of providing health education tools with accompanying graphics to provide guidance on the correct steps for caregivers in the operation of nasogastric tubes. The cost of learning nasogastric tube care using dummy people is high; therefore, it is necessary to develop cheaper simple health education tools in clinical nursing practice to enhance the quality of nasogastric tube care and prevent accidents involving physical or psychological damage to both patients and caregivers [42]. In the process of home care, foreign caregivers encounter problems such as the language barrier or an inability to remember the operation steps, and some of them consider that the operation steps are not important. To solve these problems, nurses use methods such as operation videos and health education leaflets to improve the efficiency of communication [43]. In addition to these tools, the effectiveness of learning has a relationship with the frequency of the nurses visiting the home care, the operation specification, and the caregivers’ health literacy. There is a need to establish a complete set of policy measures simultaneously, through different health education tools and learning methods with regard to intervention in the feeding process. Those interventions demonstrate greater accuracy in the use of the nasogastric tube in nursing care [44]. Graphical information can improve the carer’s understanding of patients’ medication and medical information. It is recommended that graphic designers should be included in the medical field; however, the use of graphics should be adapted and verified by users of different cultures [45].

In this study, nasogastric tube operation was taught to home caregivers at home by example. Combining the actual work needs of nurses, professional designers, and graphic designers provided research cooperation and actively assisted in the graphic recording, providing a relatively standardised development process. Home caregivers need to be able to operate independently for the majority of the time without the guidance of nurses; appropriate health education tools can help home caregivers to recall the operation process in the home caring environment to correctly perform the operation.

## 3. Material and Methods

### 3.1. Research Steps and Framework

**Figure 1:**
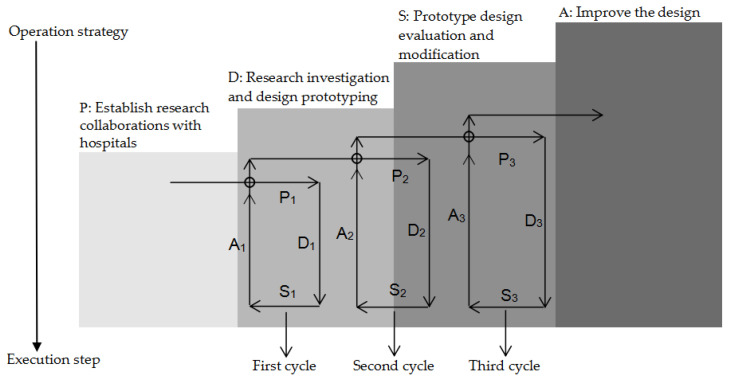
Participatory action research (PAR) steps and framework diagram.

The nurses that teach nasogastric tube home care need to spend time and energy communicating with the patient’s family and home caregivers at home. When designers and researchers join in the process, they need to constantly re-examine the problems, controllable conditions, and the cycles that can be improved in the whole operation process from the viewpoint of a challenger. In the framework of action research involving people from different professional backgrounds, PDSA carries out different operational steps at each cycle according to the research time. The circular nature of the action research promotes the development of the research and deepens the cooperation of the researchers in different fields (Figure 1). With regard to the development of graphics of nasogastric tube care in PAR, recording the research process has important significance and is an important function. The execution of the first cycle of the steps can be exploratory; researchers approach the problem from the angle of the design to explore the possibility of its operation. The second cycle of the steps is to focus on improving the existing health education tools, on the basis of incorporating the nurses’ professional knowledge into the design of the intervention. The third cycle of the steps is mainly based on the second cycle in the development of tools, combining this with the user suggestions and evaluations. Each cycle stakeholder’s degree of participation is different; the roles and the operations are different. The overall research framework was established from the early stages of the study to develop health education tools under the condition of existing medical care with the goal of using visual graphics to communicate with the auxiliary medical care, and to provide health education tools that can be used in clinical teaching.

In the first cycle, the researchers used the interview method, and different professionals expressed different views on the study process. The interview content was used to interpret the medical staff’s attitude in the process. Problems of nasogastric tube operation in home care were discovered, and relevant suggestions were provided by hospital managers, nurse supervisors, and nurses. The researchers planned and implemented the study based on the main points and suggestions raised in the interviews. 

In the second cycle, the basis of cooperation was discovered in the interviews based on the first cycle, combining the participant observation and interview and making a plan for designing the graphics of the nasogastric tube home care operation. The nurses conducted the nasogastric tube home care process. The researchers recorded the nasogastric tube home care process in the form of video audio and organised the image and text records. The professional designer made graphic designs in accordance with the recorded content. The illustrations were accompanied by bilingual text captions in Chinese/English and in Chinese/Indonesian. After completing the care process manual, the nurses reviewed the operation process, graphic content, text description, and other content, and repeatedly corrected them to improve the accuracy.

In the third cycle, nurses used the graphics developed in the second cycle to teach home caregivers. The researchers used field interviews, non-participatory observation, and final operation score statistics. After the consent of the nurses and the patient’s family had been obtained, the patients were selected randomly and recruited for the clinical operation test. This study passed the National Cheng Kung University Human Research Ethics Review Committee Version (106-149). Informed consent and signatures were obtained from the subjects, and the nurses evaluated the home caregivers’ operation process. According to the results presented in the first and second cycles, the nurses used an A4-size nasogastric tube graphic manual to instruct the home caregivers on the operation. Based on the feedback on the description content of the graphics, the foreign home caregivers corrected the Indonesian translation. Evaluation forms were designed according to direct observation of the procedural skills of Mackenzie Hospital. Nurses assessed the scores of the home caregivers’ operations.

### 3.2. Participants

In total, 18 participants (Table 1) were involved in this study, including six participants with a design background (G1). Researcher R01 proposed the study based on their experience of previous studies, and the G1 team worked together to complete the study. Among the participants, R06 was a professional designer with senior experience in information graphics and graphics who could skilfully use the drawing software and accurately draw the graphics of the operation steps. There were five participants with a medical background (G2), including a nursing supervisor who conducted ethical practice and supervision of the research. Two nurses participated in the whole process from the beginning to the end of the study. There were seven home caregivers who finally used the health education tools (G3). This study did not have specific requirements with regard to the caring experience of the home caregivers. To ensure the privacy of the participants, all the participants were given codes and concealed key words such that the basic information content could not be guessed but the authenticity of the research was not affected.

**Figure 2:**
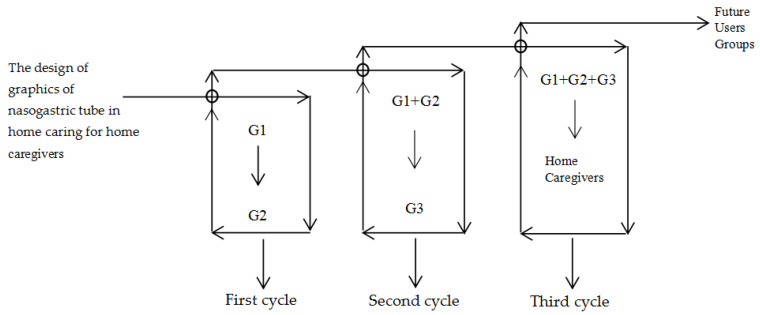
Target participants group structure diagram.

In the first cycle of the PAR, the G1 group led the research for the other participants and selected an appropriate hospital for cooperation. G2 entered the phase of joint research. In the interview process, the medical personnel were equally involved in the dialogue and determined the nature of the cooperation. In the second cycle, the G2 group was the main participant. The G1 group recorded the operation of the G2 group and made graphic drawings. Both groups repeatedly confirmed the operation content and the graphic content. The G2 group asked the G3 group about its willingness to participate in the study, and G1 together with G2 invited G3 to participate in the third step. In the third cycle, the G3 group was the main participant but G1, G2, and G3 were co-participants: G3 in the role of the learner and operator; G2 in the role of teaching the nasogastric tube steps to the home caregivers, correcting action, and sorting; and finally, G1 summarised the findings and developed the graphic printing health education tools to be used in nasogastric tube home caring in the future (Figure 2).

### 3.3. Research Tools and Data Processing

Based on the differences in the participants’ professional fields and the division of labour, PAR was used as a research tool. The participants clearly defined the purpose of their action, and their rights and interests were guaranteed in the process of participating in the research. The layperson has obvious limitations in participating in scientific research, and usually the participants’ actions are guided towards the researcher’s ideal scope. Therefore, in PAR it is necessary to combine the actions of experts and laypeople [46]. The use of PAR in this study will enable design professionals to understand the processes and methods of healthcare nurses, as well as enable nurses to better understand the professional assistance that designers can provide for them. When cooperating with the nursing community, designers need to confirm what is being designed. For home caregivers who are responsible for patients, the procedure and details of nasogastric tube operation are very important and may affect the interests of the patients. Using the PDSA quality management method can confirm each cycle of the action research: in the first cycle, the feasibility of the research problems are confirmed; in the second cycle, the health education tools’ content is repeatedly confirmed; and in the third cycle, home caregivers in operation and feedback provide suggestions repeatedly. Therefore, in the three cycles of the action research framework, the operation of each cycle should be confirmed by the participants, and the processes of planning, operation, learning, introduction and implementation should be clarified by PDSA. The health education tool suitable for nasogastric tube home caring should be jointly developed for the reference of home caregivers.

### 3.4. Research Limitations

This research required human research ethics approval from the institute and the hospital. The research needed completion of the survey to be achieved within a specific time and also required the cooperation of the hospital nurses and the consent of the patient’s family. The final evaluation process also required the consent of the home caregiver, and the nurse asked the home caregiver to give their informed consent with a signature.

After sorting out the number of nurses’ individual cases, the nurses told the researcher which of their cases could participate in the evaluation of the study, which required the researcher to enter the patient’s home with the nurses. In addition to giving their consent, non-nursing professionals must operate according to hospital schedules. Due to the difficulties in obtaining actual test samples, the researcher originally planned to recruit more than ten groups of participants. After on-the-spot inquiry, the researcher excluded cases such as the family members of patients who did not agree to cooperate with the study or did not use a nasogastric tube. A total of seven groups of cases participated in the survey.

## 4. Results

### 4.1. Cycle 1: Make a Plan for the Graphics Development of Home Caregivers

At the beginning of the study, the researcher proposed to solve the problems related to the process of home care by means of graphics, but the whole problem was not clear. The designers needed to find partners with a medical background, reach a consensus with the medical professional researchers (P1), and write the research plan (D1). The teams of both groups were able to accept each other’s suggestions, initiate applications for in-hospital research applications and human ethics reviews (S1), and establish an agenda for the implementation of the research plan (A1).

Researcher R01 had a long-term focus on the use of graphic design to solve the problem of doctor–patient communication, discovering graphical intervention in home care and helping caregivers in their understanding and memory of the operation process. However, the quality of the graphics used as health education tools need to be improved and should include graphic accuracy and evaluation to promote learning, teaching, and memory. In discussions with nurses, the researchers found that when local home caregivers were being taught, the new foreign home caregivers had trouble communicating because of the language barrier, making it difficult to teach them complex home care work. After the theme of the plan was established, the main researcher, R01, first formed a team in the field of design, establishing a research team with researchers in the design background (R02, R03, R04, and R05) and searched for a suitable hospital to prepare their proposals and propose their intention of cooperation. After the hospital groups agreed to cooperate, five study participants I01, I02, I03, I04, and I05 participated in the cooperative discussion, forming a research group with a medical background; among these participants I01 was the main manager of the hospital and I02 was the nurse supervisor. The common characteristic of the two groups was that each participating group had a person in charge who was responsible for communication of the research issues, the meeting time, research plan, and preparation of the research materials. In the first part of the participation process, the heads of the two groups communicated with each other and with the other participants and explained the research matters. The hospital had a positive cooperative attitude. The hospital believed that the foreign caregivers experience communication problems in carrying out home care. The hospital was willing to provide assistance to those who needed the hospital’s cooperation in this process. In the course of their communication, the two groups hoped that the research plan could be put into practice. As a result of past experience, the hospital had doubts about the graphic carrier and the specific application of the flowchart proposed by the researcher. As for the implementation of the study, the hospital administrator (I01) believed that:

*During the implementation of the design, user convenience should be taken as the centre. For example, previously during the use of the hospital registration system, the registered machine was purchased but it could not be further promoted due to the problems in the operation process, resulting in the loss of the hospital after the purchase of equipment. In the process of cross-discipline cooperation, effective designs should be made to help solve the needs of patients, doctors, and nurses. Otherwise, if all the designs cannot be applied, there will be no good cooperation cases or the hospital will suffer losses.*

**Figure 3:**
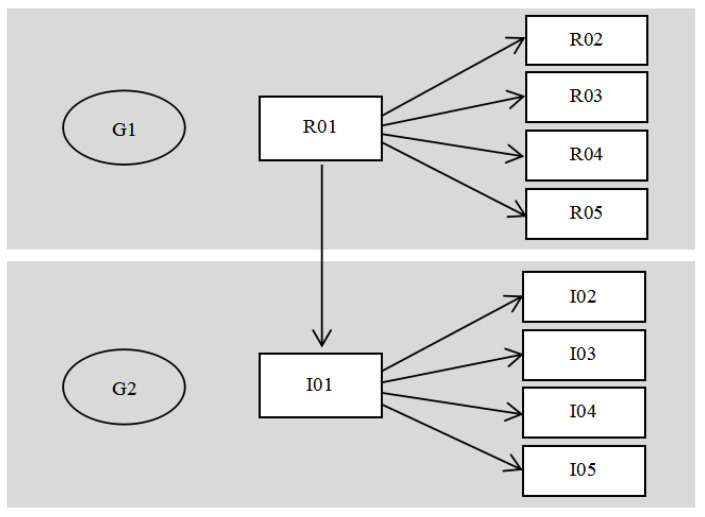
Participants in the first cycle.

According to the researchers, the form of the health education tools can be adjusted according to the clinical operation needs of the medical staff. The graphics drawn to represent the home care steps should have a standard design process, and the graphics should be optimised step by step so that home caregivers can learn or recall the operation steps according to the graphics. Participant G1 proposed the study using his own speciality as the starting point and decided to develop a graphic tool for nasogastric tube home care, aimed at helping G2 and G3. G1 invited G2 to join the research and proposed the scheme of cooperative research (Figure 3). Participant G2 believed that G1 needed to address the real issues in nursing and comply with the relevant medical ethics. At the early stages of the study, the decision makers communicated repeatedly with the hospital administrators and confirmed the problems, proposed a research case whose main objective was the design and application of the graphics representing the home caring process of the nasogastric tube, and then applied for the human ethics review. After the research proposal and the application for the human ethics review were approved, the researchers communicated with the hospital to apply to execute the research case. The participants with different research backgrounds were required to communicate continuously and plan the expected research schedule of cycle 2, cycle 3 and the expected output of the research results.

### 4.2. Cycle 2: Design and Revision of Nasogastric Tube Home Caring Procedure Graphics

Based on the design of the nasogastric tube home caring procedure graphics plan, the nurses proposed a suggestion for the study (P2). Nasogastric tube care in home nursing is mainly divided into cleaning and feeding. The nurses believed that in the process of home caring, the caregivers mainly carry out external operation nasogastric tube inspection of the tube touching the skin, as well as carry out the specification of feeding. Preparation of the tool, inspection, implementation, and precautions should all be reflected in the graphics. In the second cycle of the PDSA implementation process, video records were made mainly for the nasogastric tube caring process, and the nasogastric tube home care process step graphics were completed (D2). Repeated revisions were made between participants G1 and G2 (S2), and nasogastric tube home care procedure graphics were created that were fit for the nurses’ use (A2). 

**Figure 4:**
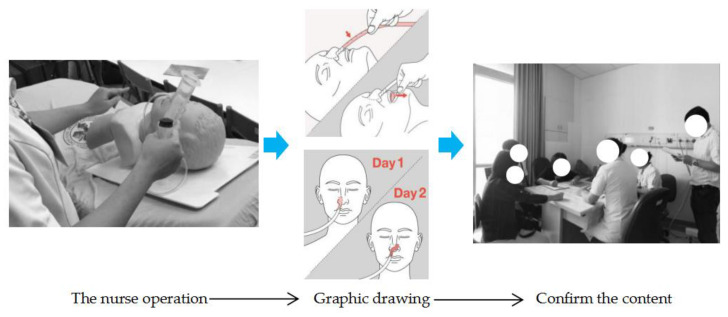
Transformation and content confirmation of nasogastric tube home caring graphics.

Nurses thought that using actual patients for the operation at the early stages may raise ethical issues because of the recording and production. Therefore, it was decided to implement the step of the recording procedure using the medical dummy model. The nasogastric tube home care demonstration operation was taught by two nurses I03 and I04; a design researcher, R02, recorded the video, and four design researchers, R01, R03, R04, and R05, were participatory observers. The nurses taught the nursing procedure, the researcher continued with the operation exercise separately, and then the nurses corrected the action and the specification, revealing the unclear action procedures that needed to be confirmed with the nurses on the spot. The researchers divided the video recordings into words and captured images, and the nurses confirmed the accuracy of the content with regard to nursing materials and procedures. Three researchers, R02, R03 and R04, collected the key images and organised the operating steps into a table; these steps were mainly divided into the nasogastric tube cleaning steps and the feeding steps. After the data had been sorted out, the nurses confirmed the content, including the accuracy of the words and sentences and the image selection. The data were reviewed and classified according to their attributes. The preliminary graphic design was conducted after researcher R01 reviewed the data after it had been completed. R02, R03, R04 successively tried to use the whole-body line graphic, the simulated human graphic, different thickness line graphics, and other forms of graphic design. Too fine a line may result in the user not being able to accurately identify the graphic according to the design group discussions, and it was thought that the operation graphics should adopt line and colour in their expression, and the nasogastric tube parts and the body’s performance should match the symbol. Researcher R06 carried out the complete design and drawing to ensure a uniform graphic style; researcher R01 led the team to continue when the design of the image content was confirmed. After the design draft was completed, the G1 team submitted it to the G2 team for review, adjustment, standardisation of the words, accuracy of the sentences, confirmation of the accuracy of the drawings depicting the nurses’ actions, and modification of the graphics by carrying out the repeated correction process of ‘Design–Fix–Redesign–Re-fix’, including confirmation of the details of the graphics (such as the rotation of the nasogastric tube in graphic should be annotated; the nasogastric tube needed to show the change of the position of the gummed tape on the first and second day). The nurses thought that A4 size for the health education tools for home care would be easy to find and read, and so the designer group used this requirement for the design and the making of the prototype (Figure 4).

**Figure 5:**
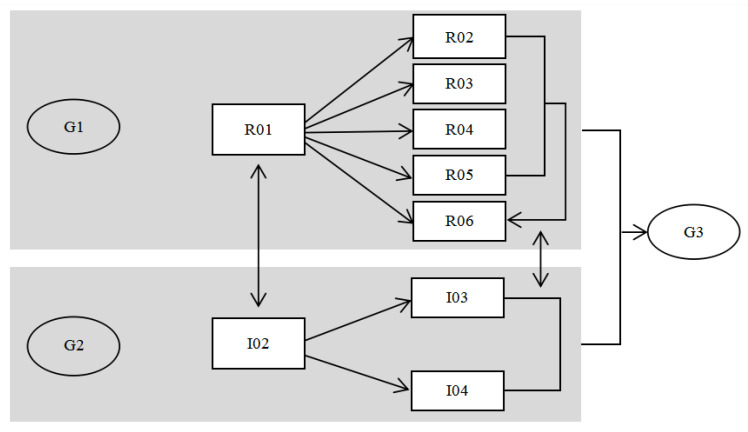
Participants in the second cycle.

Researcher R01 led the G1 group, the G1 group cooperated with the G2 group, and the above research process was supervised and implemented by the nursing supervisor I02. To ensure the smooth progress of the study, the G2 group was responsible for the execution and operation of the participants during the study, and the G1 group was responsible for the steps such as the recording and learning of the operation procedure, the drawing of the graphics, the confirmation of the text details, and the selection of the graphic style and the correction. The main leader in the participant groups was responsible for monitoring and correcting the rationality of the implementation steps of the research operation (Figure 5).

The operation of this cycle focused on the repeated confirmation of the operation steps and the graphics to ensure the accuracy of the nursing operation content. The design group, G1, was taught by the medical group, G2, and continuously learned from and communicated with the nurses until the nurses considered that the graphic content drawn in this cycle could be used. As the dummy model was used in the demonstration operation, some real operation situations could not be represented, resulting in the loss of operation steps. The graphic correction was made based on the daily operation experience of nurses and the daily situation of cases to show the operation position more clearly.

To complete the design, communication with the nurses led to several revisions. The first amendments were: (1) add a new ‘the nasogastric tube turns in half a circle’ graphic and correct the ‘need to change the sticking position of the nasogastric tube every day’ graphic, as the designers had wrongly misunderstood the ‘need to change the sticking position of the nasogastric tube every day’ as the ‘need to change the position of the nasogastric tube every day’. The researchers found that in the video recording the observers had no record of this part of the subtle operation; therefore, ‘the nasogastric tube turns in half a circle’ did not appear in the action records relating the images to the text; (2) redraw the graphic of ‘after the feeding is completed, turn the tube back, then pull out the syringe to end the feeding’ and add the graphic of ‘cover the tube and clean the empty syringe’. The reason for the correction was that the reflection of nasogastric tube had been drawn incorrectly. Modifications were made as to the correct folding direction of the nasogastric tube and the addition of the injection tube. The nurses also suggested that the procedure of drawing out the tube core and cleaning it should be described in detail. The second correction was the addition of a graphic of ‘the patient should be in a half-sitting position during the whole feeding process’. The nurses thought that the home caregivers were more likely to forget this step from the actual teaching, so the graphic was added to facilitate their review of home caring (Table 2).

### 4.3. Cycle 3: Developing Sample and Evaluation of Graphical Health Education Tools

After the nurses confirmed the operation procedure graphics of the nasogastric tube in home care, the health educational tool was output by the colour laser printer for the clinical user test, and the nurses were responsible for the user test and for the evaluation of the health education tool so that the designer could improve the final design (P3). The nurses used the graphical health education tool to teach the caregivers, and then the nurses evaluated the caregivers’ operation (D3) and asked the caregivers to give feedback on the graphics and the text descriptions (S3). Finally, the researcher and the nurse discussed the content and then corrected it (A3). The process was also repeated and verified in accordance with the PDSA implementation steps to ensure that the final nasogastric tube home care operation steps, graphics, text, and translation were correct and effective.

**Figure 6:**
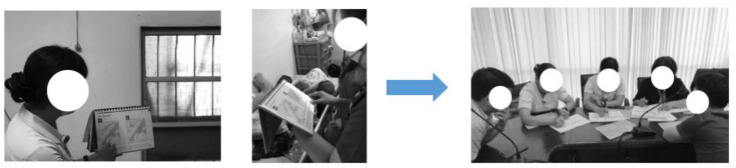
The nurse provides graphic evaluation for the home caregivers, and after the assessment there is a meeting to discuss the modification of the graphics.

The specific operation process of the third cycle was mainly divided into three steps: (1) the nurses found caregiver participants suitable for evaluation, and the caregivers needed to agree and sign the informed consent (Indonesian and Chinese version) to participating in instruction and evaluation of the operation graphics. The researchers were required to conduct non-participatory observation in conjunction with the nurses’ visits to the patients; (2) the nurses taught the home caregivers the nasogastric tube home caring process. The operation steps and precautions were explained and demonstrated with graphics, and then the home caregivers performed the operation and the nurses checked the effectiveness after the care procedure had been completed; (3) the nurses evaluated the home caregivers, completed the final evaluation form, and asked the caregivers to give relevant suggestions on the health education tools. After the completion of the above three steps, the researchers of the study convened a meeting to revise the graphics of the final nasogastric tube home care procedure (Figure 6).

**Figure 7:**
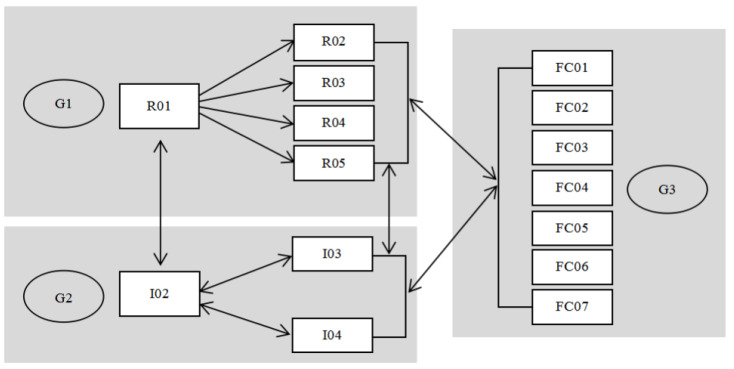
Participants in the third cycle.

Two nurses, I03 and I04, participated in the final application evaluation. The communication process between the G1, G2, and G3 groups was carried out under the supervision of the nurses’ supervisor I02. After cases of family members’ disagreement were excluded before the end of the study, seven cases (FC01–FC07) were included to form the G3 group for assessment use. Among them, six foreign home caregivers and one patient’s family member attended the evaluation of this study. R01, R02, R03, R04, and R05 of the main researcher team G1 observed and recorded using a non-participatory observation method. G2 needed to repeatedly confirm G1 and G3 and was also responsible for the communication between groups G1 and G3. However, due to the long evaluation cycle, the researchers in G1 needed to repeatedly encourage evaluation. Since the graphics were still in the development stage, the daily workload of the G2 group increased. G2 was willing to continue the study in order to obtain the research results of the health education tools (Figure 7). The operational performance evaluation of the home caregivers was divided into caring preparation, nasogastric tube cleaning, and feeding procedures; nurses also conducted health education tool study effect evaluation. For this part of the evaluation, the total score was six points, scored from the average calculated in the table for each item, including 1–2 points as ‘failed to meet the evaluation standard’, 3 as ‘close to the standard’, 4 as ‘reached the standard’, and 5–6 as ‘exceeding the standard’. In the evaluation table, the nurses and home caregivers were given satisfaction scores for this study, which ranged from 1–10, indicating satisfaction from low to high. In addition, observation time and feedback time were also recorded in the evaluation table to reflect the subjects’ operational execution time and degree of proficiency (Table 3).

Assessment found that some caregivers had caring experience, but the operation process did not follow the normal standards of correct implementation. Since most of the home caregivers were foreigners, their communication with nurses was not smooth. In conjunction with the graphic manual, the home caregivers showed that they could understand the operation in the graphics and correct the translation of the graphics description. The nurses would use the graphics again to engender caring in the process of the operation and describe the contents and steps of the graphics in detail, so that the caregivers were clearer about the matters needing attention in the process of caring at home.

### 4.4. Combined with PDSA’s Introspection on Participatory Action Research

In the existing cross-disciplinary cooperation, the main researcher usually takes the lead and the role of the other participants is weakened. In the PAR, participants are gradually added to each cycle of the study, and they can fully express the issues to be studied in each part of the study. The interactive roles of the participants are changed and the leadership roles are fluid. Considering the research content, G1 was the leader of the study in the first cycle, G2 was the leader of the study in the second cycle, and G3 was the leader of the study in the third cycle. Leadership awareness and teaching strategies have been proposed through cross-disciplinary cooperation. Although the researchers believe that doctors and nurses worry about novice operations as obstacles to teamwork, cross-disciplinary cooperation can strengthen their strengths and make up for their weaknesses [47]. The researchers with a design background initially played the role of organising and planning the research, and then the role of participatory operation and evaluation in the second and third cycles. Participants from a medical background initially played a role of passive cooperation, and later had important roles in strategy formulation and operation. In the second and third cycles, these participants mainly confirmed the accuracy of the professional operations. Although the home caregivers were not involved in the formulation of the strategy, in the third cycle the home caregivers could directly report on the rationality of the formulation of the PAR strategy and the effectiveness of the implementation.

The basic model of the study was the process of using the PDSA during operation after performing the first cycle, and then forming the second and the third cycle operation plan. In the structure, from planning to implementation, most of it is at the strategic level, and the decisions made are generally determined by the possessive resource owner. The difference between participatory research and traditional research lies in the power of the participants in the research process [48]. In the first cycle, researchers from a design background put forward the need for cooperation because they believed that they could help the medical side solve the corresponding problems by means of graphics. The designers thought that the graphical method was important and could play a leading role in the research, and they proposed the graphical strategy to solve the problems of the home caregivers. In the second cycle, when the nurse participants had entered the study, the design professionals and caregivers reached a consensus that the operation strategy was important. The researchers needed to obtain the nurses’ professional assistance to complete the relevant design, and so the designers, in accordance with the nurses, put forward a strategy to solve the problem using video recording as a method of operation. After completion, the graphic contents needed to be confirmed by the nurses before entering the next cycle evaluation; therefore, in this part, the nurse study participants held a dominant position and had an important role. In the third cycle, the researchers and the nurses formed a joint strategy to evaluate the home caregivers; the home caregivers needed to agree to evaluate its operation. The home caregivers evaluated the health education tools and provided relevant advice according to their work experience and their number of years of experience; therefore, verification of the home caregivers location was important.

Professional respect is very important for the establishment of cross-disciplinary cooperation. In the spiral structure of PDSA PAR, the three participating groups continuously confirm the professional knowledge content transmitted to confirm the level of understanding. During the graphic design stage, it was necessary to cooperate with the medical staff who could assist the designers in revising the design drawings. The degree of cooperation and joint participation are key factors. Cross-disciplinary groups must have a deep understanding of each other’s industry values and methods of solving problems, and learning from each other will become the motivation to eliminate stereotypes and cooperate with each other [49]. Implementation of PAR, on the one hand, controlled the accuracy of the graphic design and, on the other hand, the participation of the nurses, home caregivers, and researchers in the content and revision of the study to improve understanding. The home caregivers said that although some operations still needed to be memorised, the use of the graphics improved the previous situation of home caregivers having to repeatedly ask for the nurses’ caring knowledge. The graphical health education tools can recall the operation process of home caregivers. The spiral structure of PAR constantly uses PDSA to correct deviations from the research goal, so as to ensure the accuracy of the research results.

## 5. Conclusions

This study explores the cooperation between different stakeholders under the framework of PAR and uses the form of PDSA to conduct research quality management. This research was initiated by designers, and the overall research planning was carried out on the design, production, and evaluation of the graphics. The researchers with a design background promoted the process of research design, standardised the operation cycles of the graphics development and design, and established the cross-disciplinary cooperative relationship. The spiral structure of PDSA PAR was used in the study to solve different research problems according to different research cycles and complete the final research goal. In the process of intervention in the field of nursing, design researchers are looking for a relatively universal design method to make graphical drawing more in line with the needs of healthcare users.

In the process of developing medical process graphics, it is necessary to consider the enthusiasm of the participants to cooperate with participants from different professional fields, plan clear goals in the research process, and ensure that the participants are also leaders in each cycle, jointly formulating strategies and sharing the research results. In this study, the accuracy of graphic development of the health education tools was improved, the process of learning and evaluation of the graphics was added, and the accuracy of the graphics was constantly modified in operation to ensure the quality and rationality of the graphic development. The feasibility of using graphics as a communication aid or health education tool deserves further study. Simultaneously, the scope of the results of this study needs to be promoted and compared with other forms of nasogastric tube home caring methods or tools, such as health education leaflets, oral instruction, and video operation. The clinical teaching effect tests should be statistically compared using quantitative methods. Additionally, this study is an exploratory operation into cooperation between the design field and the health care field to provide a reference for the graphical development method of medical care operation processes.

## Figures and Tables

**Table 1:** Interview participants’ background and research implementation.

Participant Groups	Participant Code	Gender	Age	Working Experience: Year	Participate in the Division of Labour and Work Background
Participant groups G1: design background, researcher)	Researcher-R01	Female	50–60	Design (>30)	Establish cooperation and liaison with hospitals, establish research plans, interview, observation and design guidance.
	Researcher-R02	Female	20–30	Design (>7)	Participate in interviews, records, verbatim manuscripts, home care assessment, text writing and collation, data consolidation, design graphics manual.
	Researcher-R03	Female	20–30	Design (>7)	Participate in interviews, records, verbatim manuscripts, home care assessment, text writing and collation, data consolidation, design graphics manual, graphics correction.
	Researcher-R04	Male	20–30	Design (>3)	Participate in interviews, records, verbatim manuscripts, home care assessment, text writing and collation, data consolidation, design graphics manual, graphics correction.
	Researcher-R05	Female	30–40	Design (>15)	Participate in interviews, records, verbatim manuscripts, home care assessment, text writing and collation, data consolidation, design graphics manual.
	Researcher-R06	Male	20–30	Graphic design (>10)	Draw graphics, professional graphic designer.
Participant groups G2: 6medical backgrounds	Doctor-I01	Male	50–60	Doctor/Hospital administrator (>30)	Establishment of cooperation, hospital management, participation in interviews.
	Nursing supervisor-I02	Male	30–40	Nursing supervisor (>10)	Participate in interviews and supervise the implementation of the overall research process.
	Nurse-I03	Female	30–40	Nurse (>10)	Nursing operation, participation in interviews, correction of operation graphics, home care assessment.
	Nurse-I04	Female	30–40	Nurse (>10)	Nursing operation, participation in interviews, correction of operation graphics, home care assessment.
	Nurse-I05	Female	30–40	Nurse (>10)	Participation in interviews, correction of operation graphics.
Participant groups G3: home caregivers	Home caregiver-FC01	Female	unknown	Foreign home caregiver (>0.2)	Participated in home caring study, interview and evaluation. New home caregivers, lack of home care experience. Nurses combined with graphics teaching; home caregiver needs to learn from the basics.
	Home caregiver-FC02	Female	unknown	Foreign home caregiver (>5)	Participated in home caring study, interview and evaluation. Experienced in home care to assist in the evaluation and revision of this study. The nurse teaches in conjunction with graphics, and the caregiver provides corrections to the graphics.
	Home caregiver-FC03	Female	unknown	Foreign home caregiver (>2)	Participated in home caring study, interview and evaluation. Has home care experience but the operational details are not in place to assist this study evaluation. The nurse corrects the details of the operation by teaching them with graphics.
	Home caregiver-FC04	Female	unknown	Foreign home caregiver (0)	Participated in home caring study, interview and evaluation. The patient had never used a nasogastric tube before and home caregiver lacked experience in nasogastric tube care.
	Home caregiver-FC05	Female	unknown	Home caregiver (>5)	Participated in home caring study, interview and evaluation. At present, the family members of the patient are taking care of the patient by themselves. When there is a foreign caregiver, the family members teach them, and the family members learn from the nurse in this study to assist the evaluation and revision. Nurses combined with graphics teaching; patients’ families use the graphics teaching new home caregivers’ operation steps.
	Home caregiver-FC06	Female	unknown	Foreign home caregiver (>5)	Participated in home caring study, interview and evaluation. Skilled in home-care operation, 2 weeks of training in home care, 3 weeks of adaptation in the home environment, there will be a new caregiver to take over the work in the future to assist this study evaluation and correction. After the nurse combined the graphics instruction, the caregiver provided corrections to the graphic.
	Home caregiver-FC07	Female	50–60	Foreign home caregiver (>7)	Participated in home caring study, interview and evaluation. Specific learning time of nasogastric tube operation is unknown, but relevant experience has been gained in home care, which will assist in this study to evaluate and modify. After the nurse combined the graphics instruction, the nurse provided corrections to the graphics.

**Table 2:** Discuss additional procedures and explanations with the nurses.

Time	Before Correction	After Correction
First correction	** 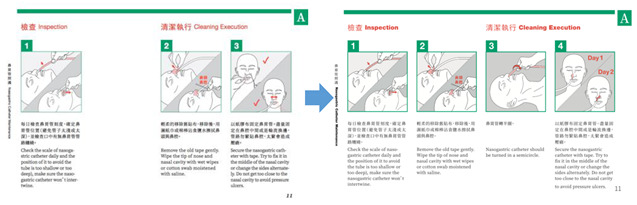 **	
	** 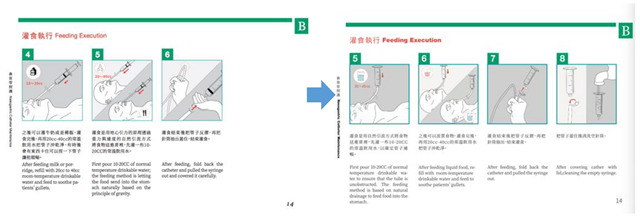 **	
Second correction	** 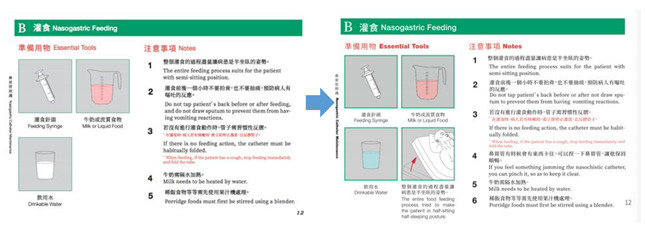 **	

**Table 3:** Assessed effectiveness of use by home caregivers.

Participants	Effectiveness	Operating Performance		Process Satisfaction		Observation Time (s)	Feedback Time(s)
		Performance (M)	Learning (M)	Nurses	Home Caregivers		
FC01	Yes	4	5	8	8	15	10
FC02	Not given	4	5	8	8	20	15
FC03	Yes	4	5	8	8	10	5
FC04	Yes	3.56	4	8	8	30	15
FC05	Yes	4.67	4	6	7	5	5
FC06	Yes	4	5	8	8	10	5
FC07	Yes	4.11	4.2	8	8	10	10
